# BioSamples database: the global hub for sample metadata and multi-omics integration

**DOI:** 10.1093/nar/gkaf1133

**Published:** 2025-11-13

**Authors:** Dipayan Gupta, Isuru Liyanage, Yasset Perez-Riverol, Enrique Sapena Ventura, Wei Kheng Teh, Tim Van Den Bossche, Senthilnathan Vijayaraja, Amnon Khen, Ugis Sarkans, Tony Burdett, Jeena Rajan

**Affiliations:** European Bioinformatics Institute, European Molecular Biology Laboratory, Wellcome Genome Campus, Hinxton, Cambridge CB10 1SD, United Kingdom; European Bioinformatics Institute, European Molecular Biology Laboratory, Wellcome Genome Campus, Hinxton, Cambridge CB10 1SD, United Kingdom; European Bioinformatics Institute, European Molecular Biology Laboratory, Wellcome Genome Campus, Hinxton, Cambridge CB10 1SD, United Kingdom; European Bioinformatics Institute, European Molecular Biology Laboratory, Wellcome Genome Campus, Hinxton, Cambridge CB10 1SD, United Kingdom; European Bioinformatics Institute, European Molecular Biology Laboratory, Wellcome Genome Campus, Hinxton, Cambridge CB10 1SD, United Kingdom; VIB-UGent Center for Medical Biotechnology, VIB, Ghent 9000, Belgium; Department of Biomolecular Medicine, Ghent University, Ghent 9000, Belgium; European Bioinformatics Institute, European Molecular Biology Laboratory, Wellcome Genome Campus, Hinxton, Cambridge CB10 1SD, United Kingdom; European Bioinformatics Institute, European Molecular Biology Laboratory, Wellcome Genome Campus, Hinxton, Cambridge CB10 1SD, United Kingdom; European Bioinformatics Institute, European Molecular Biology Laboratory, Wellcome Genome Campus, Hinxton, Cambridge CB10 1SD, United Kingdom; European Bioinformatics Institute, European Molecular Biology Laboratory, Wellcome Genome Campus, Hinxton, Cambridge CB10 1SD, United Kingdom; European Bioinformatics Institute, European Molecular Biology Laboratory, Wellcome Genome Campus, Hinxton, Cambridge CB10 1SD, United Kingdom

## Abstract

The BioSamples database (https://www.ebi.ac.uk/biosamples/) at the European Bioinformatics Institute (EMBL-EBI) is a core infrastructure resource that provides a centralized platform for the storage, curation, and dissemination of biological sample metadata. BioSamples holds sample descriptions across diverse datasets, enhancing their reusability and integrability in accordance with the FAIR (findable, accessible, interoperable, and reusable) principles. BioSamples serves as the central place to store the sample metadata for multiple repositories and archives at EMBL-EBI, including the European Nucleotide Archive, ArrayExpress, and the European Phenome-Genome Archive. In this article, we outline technical updates made to support increased sample registration and provide a better user experience. We describe four use cases whereby different communities have leveraged BioSamples to connect multi-omics data and enable the creation of public data portals and data status trackers. BioSamples is freely available, and its content is distributed under the EMBL-EBI Terms of Use available at https://www.ebi.ac.uk/about/terms-of-use. The BioSamples code is available at https://github.com/EBIBioSamples/biosamples-v4 and https://doi.org/10.5281/zenodo.17304021 and is distributed under the Apache 2.0 license.

## Graphical Abstract

**Graphical Abstract:**
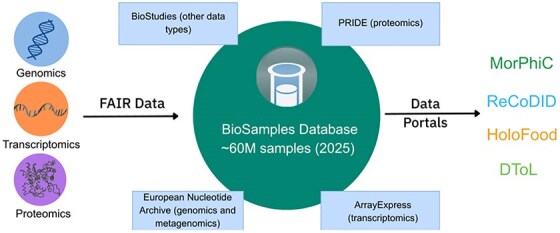

## Introduction

Registration of sample metadata at the EMBL-EBI BioSamples database [1] results in persistent identifiers (accessions) that can be used as part of submissions to other omics archives, such as the European Nucleotide Archive (ENA) [2], ArrayExpress [3], the European Phenome-Genome Archive (EGA) [4], PRIDE [5], and MetaboLights [6]. Reusing BioSamples accessions links the different data types through the metadata, providing context and enabling cross-functional analyses. In recent years, BioSamples has evolved into a central hub for coordinating sample metadata, extending beyond EMBL-EBI resources to a wider ecosystem of databases, repositories, and community-specific portals that present omics data. This expansion began in 2022 with ReSOLUTE [1], one of the first consortia to adopt BioSamples as the foundation of its sample metadata system, and has since accelerated with newer initiatives such as MorPhiC [7] and the Darwin Tree of Life project [8]. While earlier versions of BioSamples primarily supported metadata from genomics and transcriptomics studies (e.g. RNA-seq experiments), the scope has broadened substantially. BioSamples now actively collaborates with the proteomics community, positioning itself as a large-scale multi-omics hub that connects diverse resources and enables integrative analyses across data modalities.

**Figure 1.**
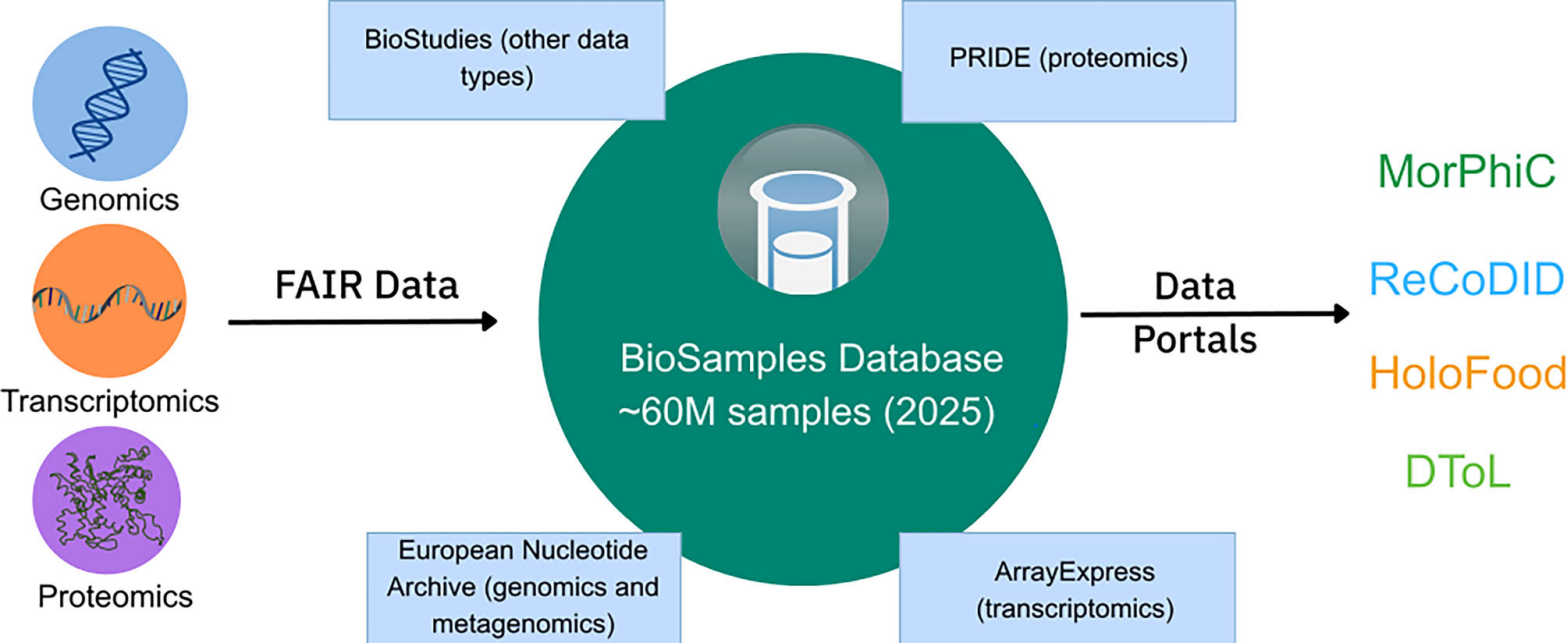
The BioSamples database links multi-omic data through its role as a central metadata hub, enabling unified dataset views via community data portals.

Since our previous NAR update in 2022 [1], the BioSamples database has seen significant growth, with the number of registered samples increasing three-fold to 60 million by 2025. In this article, we outline key infrastructure advancements, including the introduction of bulk submission capabilities to support large-scale sample metadata deposition and enhancements to the RESTful API that improve programmatic access and interoperability. Together, these developments establish BioSamples as a key hub for managing omics sample metadata in the future. We also describe the new checklist editor and the growing ecosystem of databases, resources, and portals that rely on BioSamples as their central metadata hub (Fig. 1). Finally, we highlight the integration of BioSamples with non-genomics resources such as PRIDE (proteomics), underscoring its role as a foundation for multi-omics data management and reuse.

### Technical improvements

The BioSamples team has implemented several technical enhancements to improve the database infrastructure and ensure the continued sustainability of BioSamples in response to increasing demand, as outlined below.

### Bulk sample metadata submission

**Figure 2.**
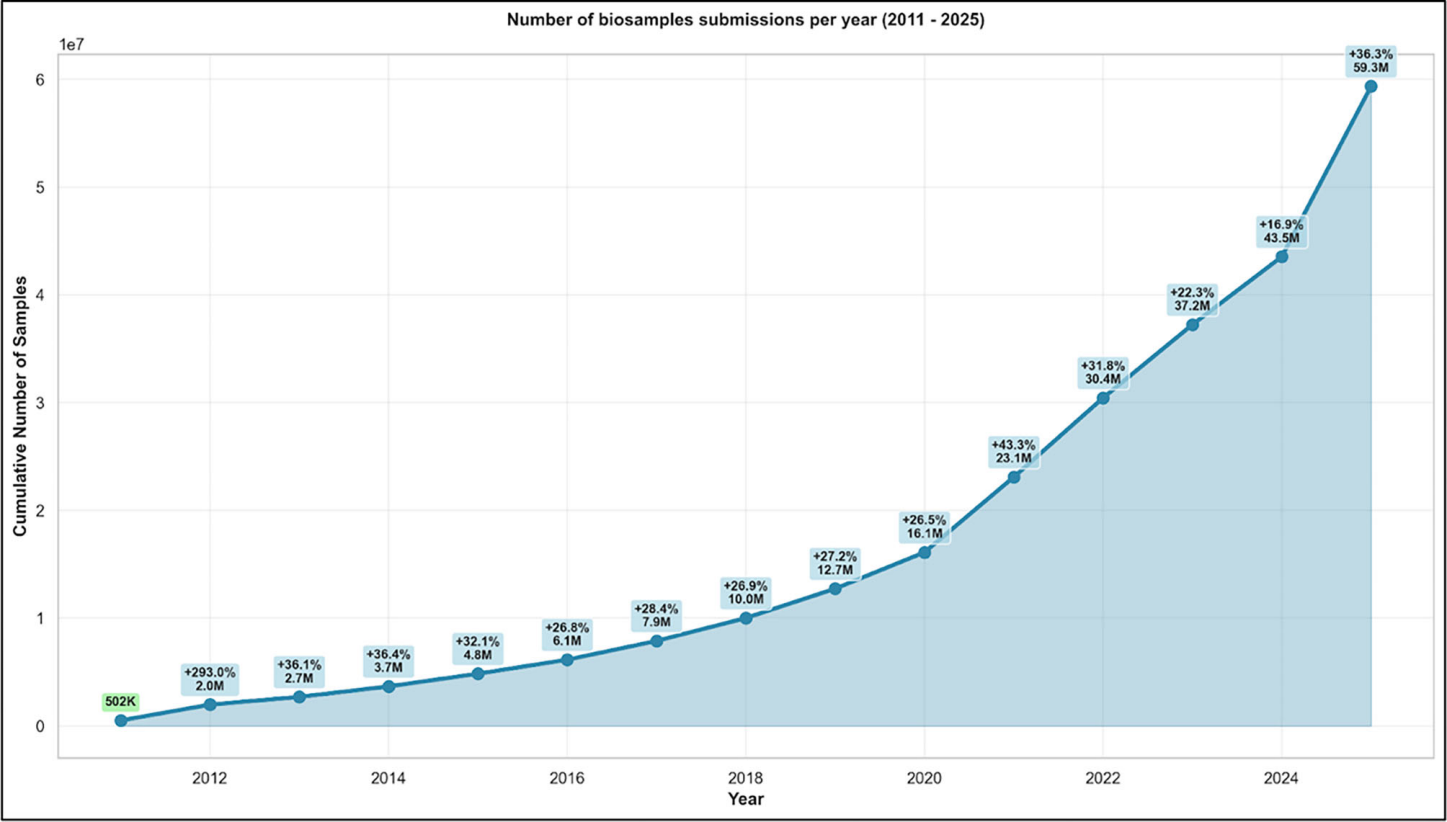
Number of BioSamples submissions per year.

Originally, the BioSamples database only accepted single-sample submissions; this approach was adequate when submission rates were modest. However, growing demands from large-scale repositories such as the ENA and consortia like the EMBL TREC (Traversing European Coastlines) Initiative; https://www.embl.org/about/info/trec/), which have increasingly delegated sample management to BioSamples, have driven a sharp rise in sample submissions (see Fig. 2). To efficiently manage the growth in samples associated with large-scale experiments and maintain the infrastructure (RESTful API, submission system) responsiveness, BioSamples has recently introduced a bulk submission framework enabling users and partner archives to submit large batches of samples in a single request. This bulk submission approach significantly reduces the communication overhead and latency inherent in processing individual submissions sequentially. By batching multiple samples, it minimises the number of request–response cycles between BioSamples and collaborating archives like ENA, resulting in faster ingestion and validation workflows. Moreover, bulk submissions help alleviate network congestion and server load, enhancing scalability and robustness. The bulk submission mechanism is supported through well-defined RESTful APIs and standardised metadata formats, ensuring seamless integration with upstream data providers and automated pipelines. This development is critical in supporting high-throughput projects and consortia that generate extensive biological sample data, enabling BioSamples to serve as a scalable central metadata hub for diverse omics data ecosystems (Fig. 1).

### Drag and drop submission

To better support researchers, particularly those without programming experience, BioSamples introduced a simplified drag-and-drop interface. Submitting biological samples one by one is often slow and prone to errors, especially in studies involving large datasets or complex designs. The new drag-and-drop upload tool allows users to submit multiple samples at the same time using a structured, human-readable, tab-delimited ISA-tab [9] format (e.g. https://www.ebi.ac.uk/biosamples/upload/downloadExampleFile). This enables researchers to prepare their sample metadata offline in familiar spreadsheet tools, significantly reducing the time and effort required for submission.

The interface also supports the creation of relationships between samples, e.g. linking a processed sample back to its original tissue/material or grouping all samples from a particular study. This helps preserve the biological and experimental context without requiring technical knowledge of APIs or ontologies. By minimising manual input and validation errors, this tool lowers the barrier to entry for high-quality metadata submission, making BioSamples accessible to a broader research community, including those in smaller labs or collaborative projects with limited informatics support.

### New checklist editor, JSON schema store, and biovalidator

The new checklist editor has been successfully developed and rolled out in 2025 by the BioSamples team, replacing the legacy ENA-maintained editor. This tool enables checklist maintainers to create and edit sample checklists along with their associated fields, offering a user-friendly interface and support for exporting checklists as TSV or JSON formats. Sample submitters can select an appropriate checklist, populate it with sample data, and submit it directly to either ENA or BioSamples, thereby streamlining the metadata submission process.

The primary goal of the new checklist editor rollout was to modernise checklist management by transitioning to a JSON-based system that is easier to maintain and extend and to enable checklist versioning. New checklists are created using the editor and stored in a centralised JSON Schema Store. Checklist versions are maintained for backward compatibility, ensuring that existing submissions remain valid even as new versions evolve.

When a sample needs to be validated, the corresponding checklist is retrieved from the schema store, and both the sample metadata and checklist schema are passed to the biovalidator [10] for validation. This integrated flow ensures consistent structure, improved data quality, and seamless interoperability with existing services, marking a significant step towards a more sustainable and flexible metadata infrastructure.

**Figure 3.**
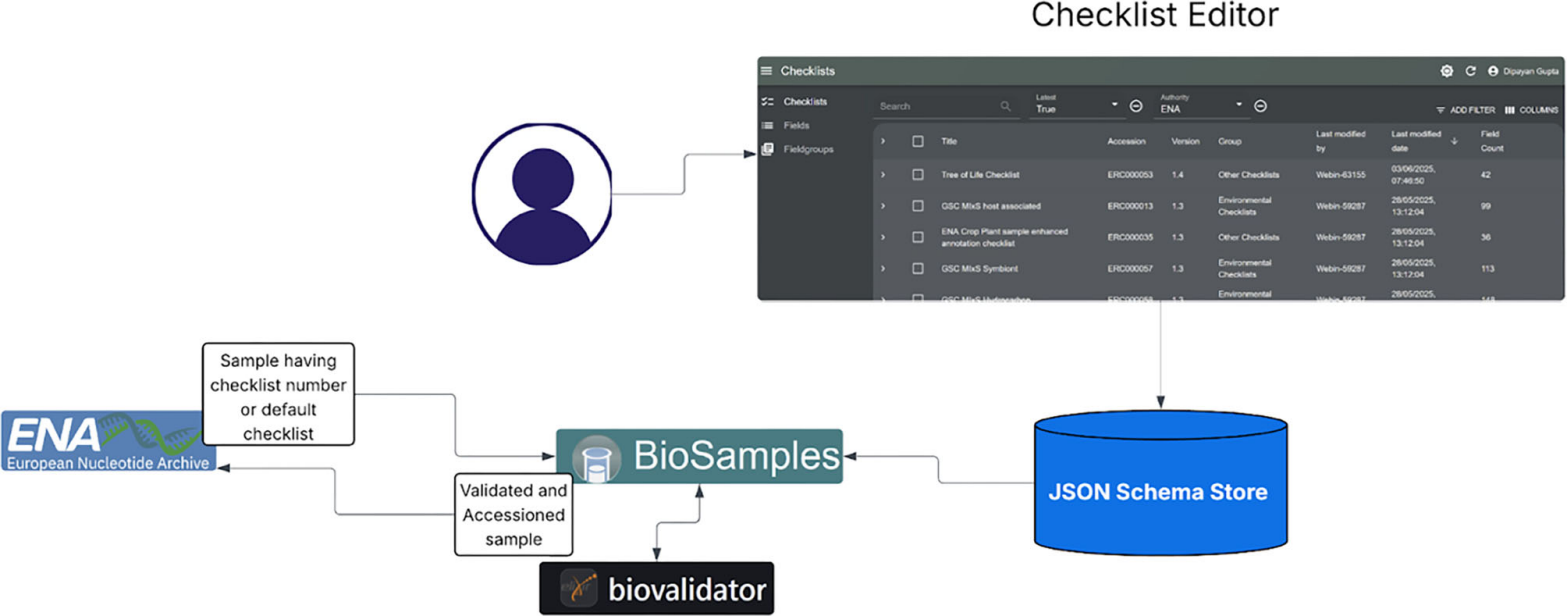
A new, user-friendly checklist editor has been introduced to streamline the internal management of checklists and JSON schemas. This enhances consistency and outsources the sample validation to BioSamples.

We have introduced new features and enhancements to biovalidator, including identifier validation, improved cache management, and support for the latest JSON Schema. These updates make the tool more adaptable for use across archives such as EGA at EMBL-EBI. Together with the checklist editor and JSON Schema Store, the biovalidator forms part of a growing ecosystem of validation tools designed to strengthen the FAIRness of life sciences metadata, as shown in Fig. 3.

### ENA–BioSamples integration

As mentioned above, the BioSamples database now serves as the central hub for sample metadata management, fully integrating workflows previously managed by the ENA. This integration covers validation, accessioning, storage, and metadata exchange with the International Nucleotide Sequence Database Collaboration (INSDC) [11] partners, providing standardised pipelines and dedicated infrastructure that ensures consistent, high-quality, and non-redundant metadata across the international data-sharing ecosystem.

For researchers, sample registration is streamlined; users can submit samples directly to BioSamples (https://www.ebi.ac.uk/biosamples/submit), independent of subsequent sequence data submissions to ENA. This separation of roles establishes BioSamples as the authoritative source of sample metadata, while ENA focuses on sequencing data. It also enables sample metadata to be publicly released earlier than the associated sequence data, a practice many large consortia now adopt for early visibility, such as the EMBL TREC (Traversing European Coastlines) Initiative. With comprehensive APIs, support for bulk and programmatic submissions and full INSDC-compliant data exchange, BioSamples provides a scalable framework for long-term interoperability, extending beyond ENA to other omics archives, including proteomics and reinforcing FAIR principles [12] for findable, accessible, interoperable, and reusable metadata.

Centralising sample management in BioSamples further enhances interoperability between repositories; the BioSample accessions registered during ENA submissions can be referenced when additional data types, such as proteomics datasets in PRIDE, are generated for the same specimens, creating a scalable pathway for linking diverse multi-omics datasets.

### BioSamples: a sample metadata hub

The deposition of rich metadata is essential for ensuring the findability, interoperability, and downstream reuse of data [12]. When datasets are deposited with minimal metadata, their utility is severely constrained; without adequate contextual and provenance information reliable analyses become challenging, and integration with other datasets is often impossible. The BioSamples database has evolved into the central repository for sample metadata across EMBL-EBI resources and is increasingly serving as a key hub for sample management within international consortia, community portals, and domain-specific databases. In this section, we describe several of these initiatives to illustrate how BioSamples can be leveraged and seamlessly integrated with omics resources, thereby enhancing the visibility, interoperability, and reusability of linked omics data.

### PRIDE database: sample metadata annotations for proteomics data

In 2021, the proteomics community introduced SDRF-Proteomics [13], a tab-delimited format designed to systematically capture sample metadata in public proteomics archives. Beyond recording sample information, the format also enables the description of the full experimental design, including details such as sample preparation, labelling strategies, and instrument settings. Because of its flexibility and community-driven development, SDRF-Proteomics has been adopted by multiple software tools as the *de facto* standard for describing proteomics sample metadata [14,15]. The PRIDE database [5], the world’s largest public repository of mass spectrometry-based proteomics data, incorporated SDRF-Proteomics as the standard way to record sample metadata during dataset submission. To ensure metadata completeness, PRIDE applies validation steps at submission based on community guidelines and standardised templates. For example, when cell line samples are used, the official cell line identifier must be provided. These community-maintained templates are integrated into PRIDE to guide researchers through the annotation process.

As part of this implementation, the first SDRF-annotated datasets submitted to PRIDE were also registered in BioSamples. Each sample annotated in SDRF received a BioSamples accession, and these identifiers were linked back into the SDRF files (e.g. https://www.ebi.ac.uk/pride/archive/projects/PXD000652). This created the first infrastructure to connect PRIDE datasets with BioSamples, enabling systematic cross-referencing of proteomics samples with other life science data resources. By 2025, >8000 proteomics samples have been deposited in BioSamples via PRIDE, positioning PRIDE as the largest non-genomic resource integrated with BioSamples. This integration not only enhances the FAIRness (findability, accessibility, interoperability, and reusability) of proteomics data but also strengthens the links between proteomics and other omics domains, paving the way for more comprehensive multi-omics studies.

### MorPhiC

The MorPhiC (Molecular Phenotypes of Null Alleles in Cells, https://morphic.bio/) project [7] is a collaborative initiative to systematically characterise gene function across human and model organism cell lines, using advanced perturbation and sequencing techniques. In one MorPhiC study, a panel of human cell lines was used to generate gene knockouts and overexpression clones for selected targets. These engineered cell lines were then differentiated, processed for library preparation, and sequenced to uncover their transcriptional profiles. To enhance the discoverability and reusability of MorPhiC datasets, curators developed automated extract, transform, and load (ETL) pipelines that parsed structured metadata from internal systems and programmatically registered each biomaterial in BioSamples using its REST APIs. The BioSamples curation API was further used to enrich sample records post-submission with cell line properties, process metadata, and ontology annotations. This API supports third-party and submitter curation, enabling ongoing improvements while retaining original submission records. Each curation event is stored with a curator ID and timestamp, ensuring traceability and version control. A public-facing, uncurated view remains accessible for audit and comparison.

To align the sample metadata with community standards, BioSamples’ built-in validation standards for transcriptomics samples were applied. BioSamples supports JSON Schema representations of community standards such as MINSEQE (https://www.fged.org/projects/minseqe/), enabling automated validation at the time of submission or curation. This ensured that key fields such as cell line, organism, protocol information, and sequencing type were present and consistent.

With these improvements, MorPhiC samples in BioSamples are richly annotated, ontology-linked, and readily searchable. Users can browse or query the project’s sample metadata through the BioSamples portal. For example, the 1600 MorPhiC sample records can be accessed directly via https://www.ebi.ac.uk/biosamples/samples?filter=attr:project:MorPhiC. These enriched metadata records are available in multiple standard formats, including JSON and XML, making them suitable for integration into automated analysis pipelines and downstream discovery tools.

### ReCoDID COVID-19 pilot study

The ReCoDID (Reconciliation of Cohort Data in Infectious Diseases, https://recodid.eu/) COVID-19 pilot study is a research project focused on collecting and harmonising multi-omics data from 151 PCR-confirmed hospitalised COVID-19 patients. To manage and share their sample metadata effectively, the project uses BioSamples as the platform to register their biological samples. Each patient is represented as a top-level BioSample record with minimum non-identifiable metadata; this then links to child samples representing different data types such as SARS-CoV-2 genome sequences (hosted in ENA), clinical-epidemiology data (in EGA), and antibody assay results (available in ArrayExpress and BioStudies). This hierarchical organisation supports participant-level linkage across diverse datasets, enabling comprehensive integration without duplicating metadata. BioSamples provides the ReCoDID project with a standardised metadata repository that ensures findability and interoperability through controlled vocabularies, ontology annotations, and schema validation. The platform’s RESTful APIs and relationship mapping allow programmatic access and browsing of linked samples, facilitating cross-repository data integration. There are currently 458 ReCoDID samples available in BioSamples: https://www.ebi.ac.uk/biosamples/samples?filter=attr:project+name:ReCoDID%20COVID-19%20pilot%20study. By leveraging BioSamples, the ReCoDID COVID-19 pilot study achieves consistent metadata registration and enhances the accessibility and reusability of its multi-centre cohort data for the broader research community.

### Darwin tree of life

The Darwin Tree of Life (DToL; https://portal.darwintreeoflife.org/) [8] project aims to sequence the genomes of 70 000 species of eukaryotic organisms in Britain and Ireland. These genomic data are important for deepening our understanding of evolutionary processes, exploring the biology of organisms and ecosystems, supporting conservation efforts, and developing tools for medicine and biotechnology. All genome sequences generated by the project are made publicly available through the DToL data portal (https://portal.darwintreeoflife.org/data?0=phylogenyCurrentClass%20-%20kingdom), which allows researchers to access and download both metadata and associated data. High-quality standardised contextual metadata were agreed upon early in the project (https://doi.org/10.12688/wellcomeopenres.17605.1). Metadata are collected and submitted to BioSamples by COPO, a meta(data) brokering system [16]. The metadata collected and submitted in the BioSamples repository enable the linking, filtering, and searching of data via the DToL Portal. There are ~67 000 DToL samples submitted to BioSamples as of August 2025; https://www.ebi.ac.uk/biosamples/samples?filter=attr:project+name:DTOL. Central to the portal is the submitted sample in BioSamples, which links raw and assembled sequences and associated data via sample relationships. A status tracker (https://portal.darwintreeoflife.org/tracking?0=phylogenyCurrentClass%20-%20kingdom) is also available to show the stage of the data generation workflow that a particular species is at. The DToL data model has also been used for the European Reference Genome Atlas [17], whose data are available from the EMBL-EBI Biodiversity portal (​​https://www.ebi.ac.uk/biodiversity/data_portal?0=ERGA&1=phylogenyCurrentClass%20-%20kingdom).

### HoloFood

The HoloFood project (https://www.holofood.eu) [18] applies a multi-omic approach, including genomics, transcriptomics, and metabolomics, alongside comprehensive metadata, to investigate how host–microbiome interactions affect animal production using different feed conditions. The aim is to improve animal nutrition, reduce environmental impact, and enhance animal welfare. All raw data, processed results, and associated metadata are publicly available through established open-access repositories, including the ENA, MGnify [19], MetaboLights [6], and BioSamples. To integrate data across these diverse domains, HoloFood makes extensive use of BioSamples, particularly its capacity to define sample relationships (e.g. ‘derived from’) and to present structured metadata tables for data types not easily stored elsewhere (e.g. histological and inflammatory marker data). BioSamples’ structured data tables allow the capture of detailed, domain-specific information in a machine-readable way. These tables are organised under a structured data section, where each entry has a type (e.g. Antimicrobial Resistance (AMR), Molecular Markers, Histology, Fatty Acids, Cell Viability, etc.) and an associated table of values. Each row in a table usually represents one measurement, observation, or tested parameter, with columns describing attributes such as the method, unit, interpretation, or result, e.g. https://www.ebi.ac.uk/biosamples/samples/SAMEA112750425. Leveraging the well-structured, richly annotated sample records in BioSamples enables the linkage and interoperability across different omics layers. This approach allows discoverability and cross-repository integration through a dedicated, user-friendly data portal (https://www.holofooddata.org/) [20] developed by the MGnify team. The portal makes use of the sample structure relationships in BioSamples to unify access to datasets across ENA, MGnify, and MetaboLights. By mapping derived data samples back to host organisms through consistent metadata relationships, the portal supports enhanced querying capabilities across all associated omics resources.

## Discussion

We have outlined several technical improvements above designed to help researchers make better use of BioSamples. In support of the sample services of large archives such as ENA and PRIDE, BioSamples is continuously evolving to handle increasing demands. To further address the increasing volume and complexity of metadata in BioSamples, we are modernising our infrastructure. The BioSamples platform is being migrated to Kubernetes to improve scalability, reliability, and manageability. In parallel, we are upgrading our search functionality by moving from Solr to Elasticsearch, delivering faster, more flexible, and more robust metadata querying. Together, these upgrades will ensure BioSamples can continue to efficiently meet the growing needs of the life sciences community. In addition, the BioSamples documentation is being updated based on user feedback collected over the past year and migrated to a new platform, making it easier for users to find guidance on submission and retrieval of samples.

In response to feedback from consortia, BioSamples is extending the types of relationships between samples to better capture experimental design. Newly introduced relationship categories, such as ‘negative control’ and ‘positive control’, are being added to the existing framework.

BioSamples is also enhancing its role as an authoritative platform for multi-omics and life sciences data by enabling external references to additional resources such as the BioImage Archive (BIA) [21]. This integration will facilitate connection of diverse data types - genomics, transcriptomics, proteomics, metabolomics, and imaging - across multiple EBI archives, improving interoperability and allowing researchers to navigate and access related datasets more seamlessly through a unified platform.

**Figure 4.**
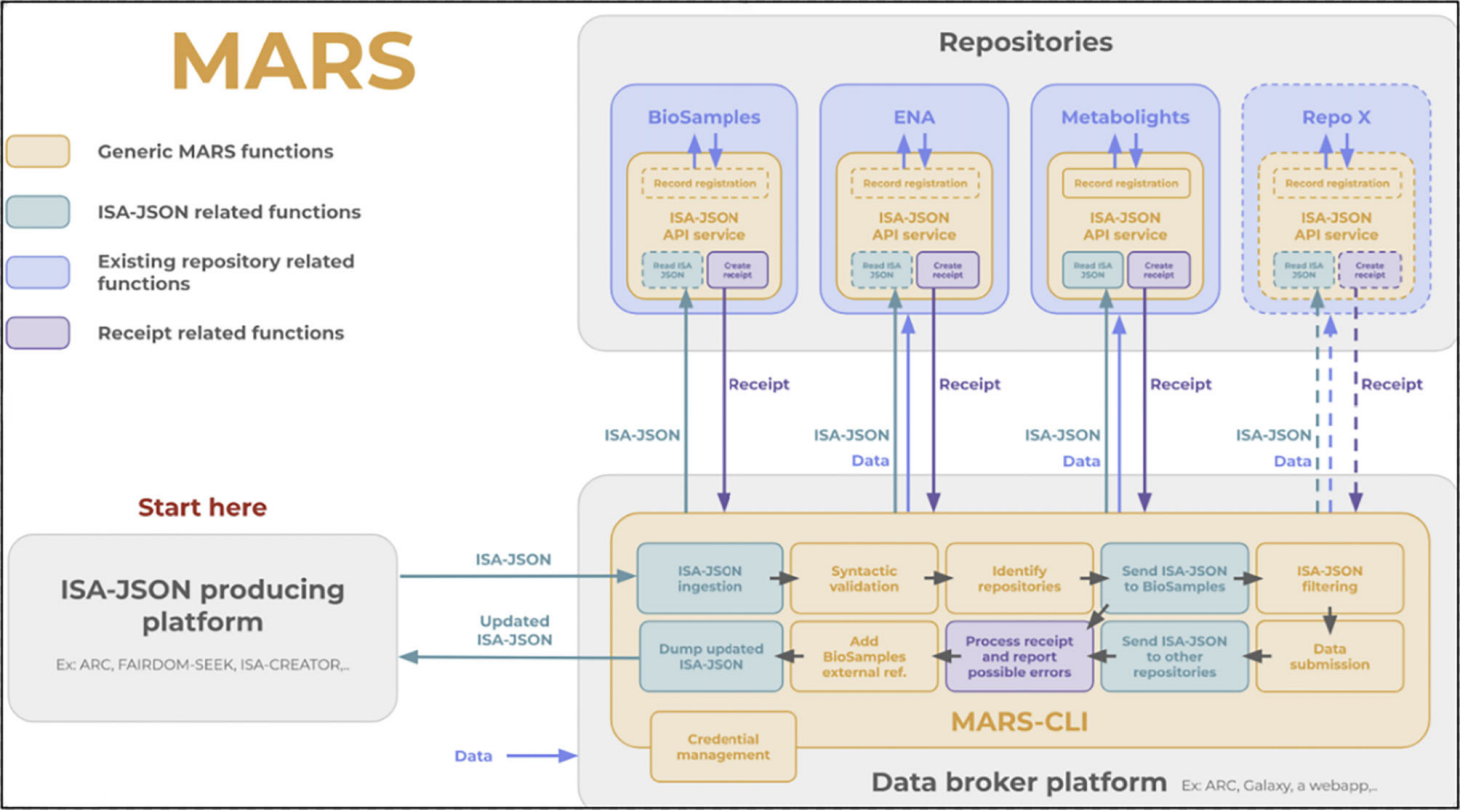
MARS data flow and use of BioSamples.

Finally, BioSamples is collaborating with the MARS (Multi-omics Adapter for Repository Submissions) initiative to streamline the submission of multi-omics data to multiple specialised repositories. MARS facilitates the use of the ISA-JSON format (https://isa-specs.readthedocs.io/en/latest/isajson.html) to standardise metadata exchange, enabling seamless data submission across various platforms, as shown in Fig. 4. Through this integration, BioSamples will serve as a main hub, linking sample metadata with other EBI archives, thereby enhancing the interoperability and accessibility of multi-omics datasets. This will contribute to better tools to enable researchers and data managers to submit, link, and manage their multi-omics experimental data more easily, aligning with the evolving needs of the research landscape.

## Data Availability

The BioSamples database is freely available, and its content is distributed under the EMBL-EBI Terms of Use available at https://www.ebi.ac.uk/about/terms-of-use. The BioSamples code is available at https://github.com/EBIBioSamples/biosamples-v4 and https://doi.org/10.5281/zenodo.17304021 and distributed under the Apache 2.0 license.

## Notes

Present address: BioFAIR Hub, Earlham Institute, Norwich Research Park,Norwich NR4 7UZ, United Kingdom
